# Optimization of multi-objective flexible scheduling for container rail mounted gantry cranes in railway logistics yards

**DOI:** 10.1371/journal.pone.0357406

**Published:** 2026-09-16

**Authors:** Huawei Wang, Chunjie Xu, Yunjiao Hou, Jiahuan Zhang, Zongyang Liu

**Affiliations:** 1 Institute of Computing Technologies, China Academy of Railway Sciences Corporation Limited, Beijing, China; 2 Department of Freight System, Beijing Jingwei Information Technology Co., Ltd, Beijing, China; Southwest Jiaotong University, CHINA

## Abstract

With the advancement of multimodal transport systems, railway logistics terminals have become critical hubs connecting different transport modes, and the efficiency of rail-mounted gantry crane (RMG) operations directly affects train handling performance. This study develops a revised multi-objective scheduling framework for flexible RMG operations in railway container terminals. The model considers three coordinated criteria: total completion time, workload balance among RMGs, and path-interference prevention under dynamic safety-distance requirements. To improve practical solvability, the framework combines resource enumeration, initial crane layout optimization, and an improved genetic algorithm with adaptive operators and tabu-search-based local refinement. In the Xi’an International Port Station case with 110 container tasks and five RMGs, the proposed IGA obtained an average completion time of 752.4 min under the baseline configuration and remained feasible under tested safety-distance and crane-number scenarios. The results indicate that integrating initial layout decisions with flexible task assignment can improve schedule quality in the tested case. The claims are limited to the reported instance, and the method is presented as a reproducible heuristic rather than a proof of global optimality.

## 1. Introduction

Under the accelerated development of comprehensive transportation system, railway container transportation has emerged as a critical component of modern logistics, leveraging its safety, environmental advantages, and high transport capacity to enhance medium-long distance mainline transport efficiency. As a pivotal hub connecting rail, road, and waterway systems, railway container terminals play a decisive role in intermodal container transshipment, where operational efficiency directly determines multi-modal transportation coordination quality and service levels. Efficient operational organization not only reduces container dwell time and improves handling efficiency, but also contributes to lowering social logistics costs and advancing green transportation transformation.

Within container terminals, gantry cranes on rail tracks serve as primary equipment for container transfer between rail trains and storage yards. Multi-gantry crane coordination aims to optimize task allocation and path planning under safety interval and equipment coordination constraints to enhance operational efficiency. Key challenges include: (1) Track resource conflicts and crane path interferences, (2) Uneven task loads, and (3) Dynamic variations in train arrivals/departures and operational requirements [1–3]. Traditional fixed-service-area scheduling, while reducing interferences, often leads to operational imbalance and resource waste. In contrast, flexible scheduling through dynamic service range adjustment improves system responsiveness and overall efficiency [4–7], but significantly increases problem complexity, transforming it into a high-dimensional discrete optimization challenge.

Facing the multi-objective, multi-constraint, and high-complexity characteristics of rail-mounted gantry crane scheduling, intelligent optimization algorithms have emerged as critical tools [8–11]. The genetic algorithm (GA) demonstrates robust global search capabilities and has been widely applied in multi-device scheduling and operational path optimization. Particle swarm optimization (PSO) achieves rapid convergence but tends to exhibit premature convergence in complex search spaces. The recently proposed sparrow search algorithm (SSA) shows balanced exploration-exploitation performance in multi-objective optimization through simulated flocking behavior. However, these algorithms still face practical limitations: (1) Most studies assume predefined crane initial positions, neglecting their impact on overall scheduling performance; (2) Standard GA encounters premature convergence and local optima issues in high-dimensional discrete spaces, compromising algorithm stability and solution quality; (3) Multi-objective coordination challenges persist, with single-objective optimization frequently substituting for comprehensive multi-objective optimization, limiting practical applicability.

To address these deficiencies, this study proposes an improved multi-objective flexible scheduling method for rail-mounted gantry cranes in railway container terminals. The approach first optimizes crane initial positioning through historical operation patterns and task distribution analysis, establishing a quality baseline for scheduling solutions. A five-tier improved genetic algorithm (IGA) is then developed, comprising: (1) task-device mapping combined with path representation encoding-decoding framework; (2) multi-criteria fitness evaluation integrating operational efficiency, load balancing, and interference constraints; (3) adaptive genetic operators with elite preservation mechanisms to maintain population diversity; (4) heuristic-based local search for solution refinement; and (5) hybrid optimization structure for global-local coordination. The IGA effectively enhances exploration-exploitation balance while mitigating premature convergence in high-dimensional combinatorial spaces, demonstrating improved solution stability and interpretability compared to standard GA.

The main contribution of this study is the integration of resource allocation, initial RMG layout, and flexible task scheduling under dynamic safety-distance constraints. Specifically, this manuscript contributes: (i) a revised RMG scheduling model that distinguishes task indices, crane indices, temporal variables, and safety-distance constraints; (ii) a resource-layout-scheduling framework in which the number and initial positions of RMGs are determined before detailed task sequencing; and (iii) an improved genetic algorithm with adaptive genetic operators and tabu-search refinement for generating high-quality feasible schedules. The method is positioned as a heuristic framework for complex terminal scheduling rather than an exact optimization method.

Despite significant progress in RMG scheduling research, existing studies predominantly treat layout configuration and task scheduling as sequential or decoupled problems, leading to suboptimal efficiency in complex terminal operations. Furthermore, most current models overlook critical operational constraints such as dynamic safety distances and equipment interference. To address these limitations, this study proposes an integrated optimization framework that simultaneously considers resource allocation, spatial layout, and scheduling, thereby filling the identified research gap in the literature.

## 2. Problem statement and modeling

### 2.1. Job scenario & problem description

We focus on container handling operations in railway terminal main work areas comprising multiple rail lines, container yards, truck access lanes, inspection gates, and control centers. Centered on multi-rail-mounted gantry crane (RMG) scheduling optimization under shared-rail continuous operation conditions, the research develops a collaborative scheduling mechanism for flexible operational zones.

Given the flexible scheduling approach adopted where operational zones for RMGs remain unassigned, potential conflicts arise from overlapping work areas during continuous rail operations. To mitigate risks of crane interference and path collisions, the scheduling model incorporates safety distance constraints [1,2,4] requiring minimum operational separation between any two adjacent RMGs at all operational phases. This constraint ensures spatial isolation between adjacent cranes through predefined safety margins while maintaining system flexibility. The proposed framework addresses the inherent complexity of multi-RMG coordination under dynamic workload distribution and real-time operational adjustments.

To systematically represent the operational structure and facilitate subsequent model development, a standardized numbering scheme is established for key components as follows:

(1) Along the railway loading/unloading line, the positive direction is defined from locomotive to train end, with sequential numbering applied to both train cars and yard column positions.
(2) In the orthogonal direction perpendicular to the railway line (positive direction away from the railway), yard row positions are numbered sequentially.
(3) RMG devices are assigned identification numbers sequentially along the train’s longitudinal axis (locomotive to train end).

**Fig 1:**
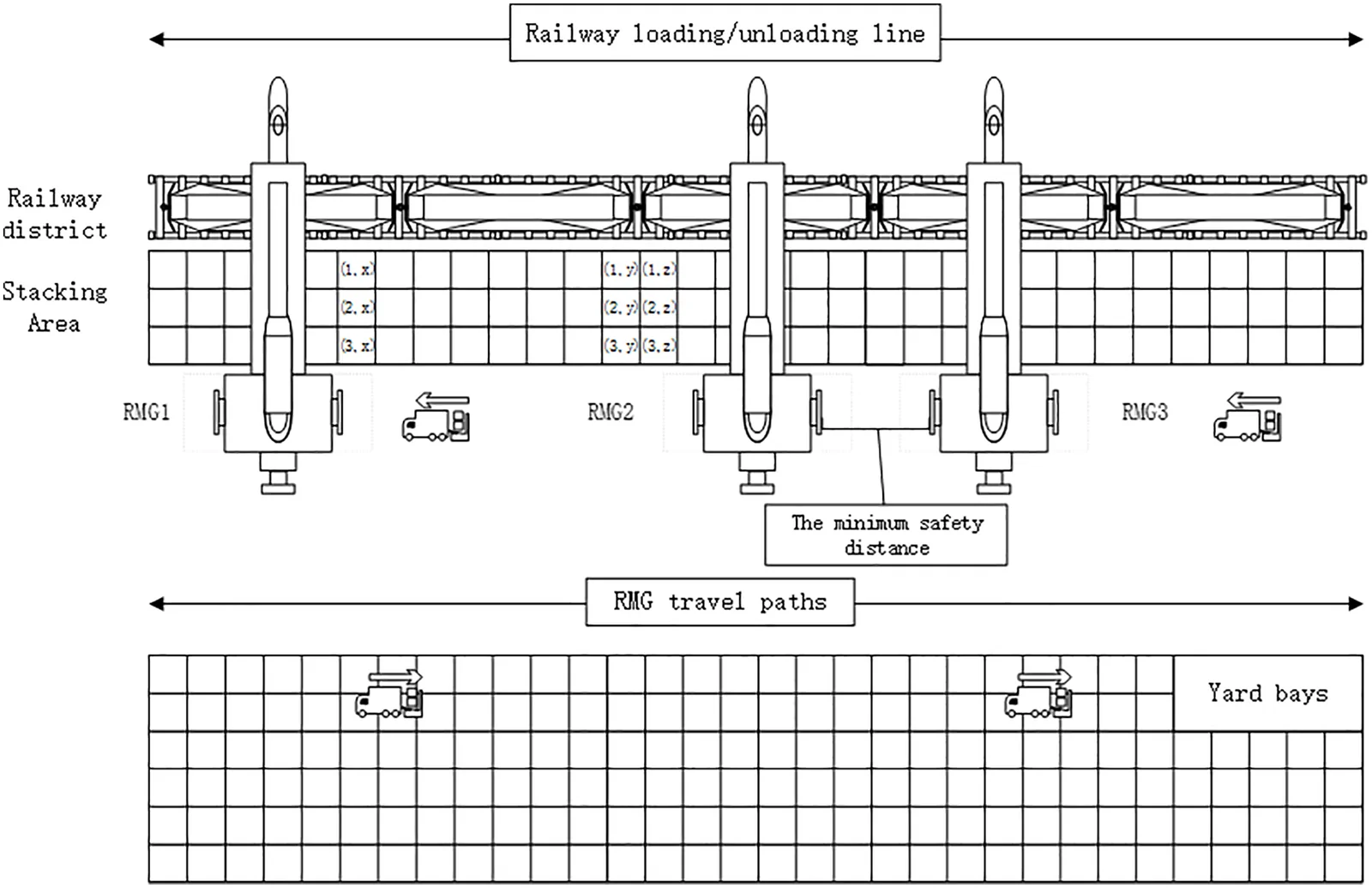
Operation scenario of multiple rail cranes.

This unified coding framework enables mathematical representation of flexible scheduling scenarios in multi-RMG railway container terminals, as schematically illustrated in Fig 1. The coordinate system integrates three-dimensional spatial positioning (train car sequence, yard column index, and yard row index) with temporal operational parameters to establish complete spatiotemporal relationships for optimization modeling.

### 2.2. Model construction

#### 2.2.1. Model assumptions.

To simplify the problem and ensure the tractability of the model, the following assumptions are made:

(1) All containers are standardized 20-foot containers (TEU) with uniform dimensions and weight, and the special handling requirements of oversized containers are not considered.
(2) The horizontal operational speed of the rail-mounted gantry crane and the vertical lifting speed are constant, and the acceleration and deceleration processes during startup and stopping are ignored.
(3) The time required for a single container grabbing or placement operation is fixed.
(4) The scheduling of tractors is idealized, and no waiting of tractors for the rail-mounted gantry crane is assumed.
(5) The rail-mounted gantry crane operates without any failures throughout the scheduling process.
(6) All task information is known prior to the start of scheduling, and no new tasks or adjustments are introduced during the operation.
(7) Each rail-mounted gantry crane can operate flexibly within the entire working area, provided that safety operational distances and non-interference constraints across tracks are satisfied.

#### 2.2.2. Symbols and parameters.

To facilitate subsequent mathematical modeling and algorithm design, this paper provides a unified definition of the key symbols and parameters involved, as shown in Table 1. This definition encompasses the critical variables related to rail-mounted gantry cranes, containers, and the yard, as well as the constraint parameters and objective functions involved in the scheduling process.

**Table 1:** Model Symbols and Explanations.

Symbol	Meaning
R	Set of rail-mounted gantry cranes, $R=R_{\text{1}},R_{\text{2}},\ldots ,R_{n}$
n	Total number of rail-mounted gantry cranes
C	Set of containers, $C=c_{\text{1}},c_{\text{2}},\ldots ,c_{m}$
m	Total number of containers
S	Set of container yard locations
T	Set of train car locations
$v_{r}$	Horizontal operational speed of the rail-mounted gantry crane (m/min)
$h_{i}$	The vertical lifting distance
$v_{l}$	Lifting speed of the rail-mounted gantry crane (m/min)
$t_{o}$	Time required for a single loading/unloading operation by the rail-mounted gantry crane (min)
$D_{safe}$	Minimum safe operational distance between rail-mounted gantry cranes (m)
$pos_{i}(t)$	Operational position of rail-mounted gantry crane $R_{i}$ at time t
$T_{comp}$	Total completion time for container train loading/unloading operations (min)
u	Total number of yard columns
H	Total height of the yard (number of tiers)
$P_{i}$	Operational sequence plan for rail-mounted gantry crane $R_{i}$
f(P)	Scheduling objective function

For the problem of flexible cooperative scheduling of multiple rail-mounted gantry cranes (RMGCS), this paper establishes a mixed integer programming (MIP) model aimed at minimizing the total completion time of container loading and unloading operations by trains, while satisfying task allocation, operational sequence, and equipment safety constraints under flexible scheduling modes.

### 2.3. Mathematical model construction

#### 2.3.1. Objective function.

To balance operational efficiency and equipment load balancing, a multi-objective weighted objective function is constructed, as shown in Equation (1):


$$\begin{array}{c} min\left[\alpha_{\text{1}}\cdot T_{comp}+\alpha_{\text{2}}\cdot \sqrt{\frac{\text{1}}{n}\sum_{k=\text{1}}^{n}(C_{k}-\bar{C}{)}^{\text{2}}}\;\right] \end{array}$$ (1)

where $T_{\text{comp}}=\underset{k\in R}{max}\{C_{k}\}$ represents the total completion time of container train loading and unloading operations (min), which is the maximum completion time of all RMGs; $C_{k}$ is the completion time (min) of the assigned tasks by rail-mounted gantry crane (RMG) $R_{k}$; $\bar{C}=\frac{\text{1}}{n}\sum_{k=\text{1}}^{n}C_{k}$ denotes the average completion time of all RMGs’ operations (min); $\alpha_{\text{1}}$ and $\alpha_{\text{2}}$ are weight coefficients satisfying ${\alpha_{\text{1}}+\alpha }_{\text{2}}=\text{1}$ and $\alpha_{\text{1}}$, $\alpha_{\text{2}}$≥0, which are adjusted according to actual operational requirements.

#### 2.3.2. Constraints.

(1) RMG Task Quantity Constraint

$$\begin{array}{c} \begin{array}{c} \sum_{k=\text{1}}^{n}\;\sum_{i\in C}x_{i,k}=|C| \end{array} \end{array}$$ (2)

Equation (2) ensures that the total number of container tasks assigned to all RMGs must equal the total quantity of containers to be loaded and unloaded in the current train operation, thereby guaranteeing no omission or duplication of tasks.

(2) Temporal Continuity Constraints
  a) Task Duration for a Single Container

$$\begin{array}{c} f_{i,k}=s_{i,k}+max\left(\frac{d_{i}^{task}}{v_{r}},\frac{h_{i}}{v_{l}}\right)+t_{o},\text{\textasciitilde{}}\forall i\in C,k\in R \end{array}$$ (3)

where $d_{i}^{task}$ represents the horizontal distance from the starting bay of container $c_{i}$ to the ending bay, and $h_{i}$ represents the vertical lifting distance. Equation (3) defines that for any RMG $R_{k}$, the completion time $f_{i,k}$ of container $c_{i}$ equals its start time $s_{i,k}$ plus the maximum of the horizontal travel time ($d_{i}^{task}/v_{r}$) and vertical lifting time ($h_{i}/v_{l}$), plus the fixed spreader operation time $t_{o}$. This formulation captures the dual-speed coupled motion where horizontal movement and vertical hoisting occur in parallel.

b) Continuous Operation Travel Duration

$$\begin{array}{c} t_{i,j,k}=\frac{\left|b_{j}-b_{i}\right|}{v_{r}},\text{\textasciitilde{}}\forall i,j\in C,k\in R \end{array}$$ (4)

where $b_{i}$ and $b_{j}$are the starting bay coordinates of containers $c_{i}$ and $c_{j}$, respectively. Equation (4) defines the travel time required for RMG $R_{k}$ to move from the termination bay $b_{i}$ of container $c_{i}$ to the starting bay $b_{j}$ of container $c_{j}$, calculated based on the bay difference and the horizontal speed $v_{r}$.

c) Time Continuity of Operation Sequence

$$\begin{array}{c} s_{j,k}\geq f_{i,k}+t_{i,j,k}-M(\text{1}-y_{i,j,k})\;\forall i,j\in \;C,k\in R \end{array}$$ (5)

Equation (5) indicates that if RMG $R_{k}$ needs to perform continuous operations on containers $c_{i}\;$and $c_{j}$, the start time of $c_{j}$ must be later than the completion time of $c_{i}$ plus the intervening travel time between the two containers. Here, M is a sufficiently large positive number, and the constraint is automatically relaxed when $y_{i,j,k}=\text{0}$. Here, M is a sufficiently large positive number, and the constraint is automatically relaxed when.

(3) Adjacent Operation Logical Constraints

$$\begin{array}{c} \sum j\in C,j\neq i\;\;y_{i,j,k}\leq x_{i,k},\text{\textasciitilde{}}\forall i\in C,k\in R \end{array}$$ (6)

$$\begin{array}{c} \sum i\in C,i\neq j\;\;y_{i,j,k}\leq x_{j,k},\text{\textasciitilde{}}\forall i\in C,k\in R \end{array}$$ (7)

Equation (6) and Equation (7) ensure that for any RMG, each container task has at most one direct predecessor and one direct successor, guaranteeing that the operation chain has no branches or loops.

(4) Single Container Single Carrier Constraint

$$\begin{array}{c} \sum_{k=\text{1}}^{n}\;x_{i,k}=\text{1},\text{\textasciitilde{}}\forall i\in C \end{array}$$ (8)

Equation (8) indicates that any container $c_{i}$ can only be assigned to one RMG, preventing the same container from being processed by multiple devices.

(5) Unique First and Last Operation Constraints

$$\begin{array}{c} \sum_{i\in C}z_{i,k}^{S}=\text{1},\sum_{i\in C}z_{i,k}^{F}=\text{1},\text{\textasciitilde{}}\forall k\in R \end{array}$$ (9)

Equation (9) ensures that each RMG’s loading and unloading task chain must have exactly one first container $z_{i,k}^{S}$ and one last container $z_{i,k}^{F}$, ensuring a clear and complete closed-loop task chain. For inactive cranes (with no assigned tasks), these constraints are automatically relaxed and do not enforce a virtual first/last task. Furthermore, the combination of Equations (6), (7), and (9) mathematically guarantees that each active crane’s task chain is a single connected sequence without any sub-cycles or disconnected branches.

(6) Non-Intersecting Operation Constraint

$$\begin{array}{c} \sum_{k=\text{1}}^{n}k\cdot x_{i,k}+M\left(\text{1}-u_{i,j}\right)\geq \sum_{k=\text{1}}^{n}k\cdot x_{j,k},\text{\textasciitilde{}}\forall i,j\in C,i\neq j \end{array}$$ (10)

Equation (10) ensures that RMGs with smaller indices must always be positioned to the left of those with larger indices, preventing crossing collisions between equipment on the same track; $u_{i,j}=\text{1}$ if and only if containers $c_{i}$ and $c_{j}$ have temporal overlap.

(7) Real-Time Safety Distance Constraint

$$\begin{array}{c} \left|pos_{k}(t)-pos_{l}(t)\right|\geq D_{safe},\text{\textasciitilde{}}\forall k,l\in R,k\neq l,\forall t\in [\text{0},T_{comp}] \end{array}$$ (11)

Equation (11) ensures that at any moment, the actual bay distance between any two RMGs must not be less than the minimum safety distance $D_{\text{safe}}$, guaranteeing that a physical safety buffer is always maintained during equipment operation. The real-time safety distance constraint and the non-intersecting operation constraint draw upon experience from recent related research to ensure that equipment maintains a safe distance and avoids mutual interference during operation [1,3].

It should be noted that although the safety distance constraint (11) is formulated for continuous time in the mathematical model to guarantee spatial isolation at any moment, the algorithm implementation (detailed in Section 3.3) adopts an event-driven discrete checking strategy. This approach samples only at equipment arrival/departure events, which are the only moments when the relative distance between adjacent cranes can reach a local minimum. Thus, the event-driven verification is strictly equivalent to the continuous-time constraint while significantly reducing computational complexity.

(8) Train Departure Time Constraint

$$\begin{array}{c} f_{i,k}\leq T_{depart},\text{\textasciitilde{}}\forall i\in C,k\in R \end{array}$$ (12)

Equation (12) ensures that the completion time of all container operations must be earlier than or equal to the train departure time $T_{\text{depart}}$, guaranteeing that the train departs on schedule.

## 3. Algorithm design and model solving

The proposed framework decomposes the problem into resource enumeration, initial layout optimization, and task scheduling. Since the number of available RMGs in the case study ranges only from 1 to 5, K is evaluated by direct enumeration. Layout optimization parameters, including the number of Latin hypercube samples, tabu tenure, restart probability, population size, generation number, crossover probability, mutation probability, archive size, stopping rule, and random seeds.

To achieve the two objectives of “minimizing maximum completion time” and “equipment load balancing” in the model, this study proposes a hierarchical progressive multi-objective optimization framework of “resource-layout-scheduling.” This framework decouples the original high-dimensional mixed-integer programming problem into two nested subproblems, corresponding to the resource allocation layer and the initial layout layer, thereby significantly reducing the solution complexity while ensuring global optimality.

Unlike traditional hybrid methods that typically focus solely on task sequencing, this study introduces a three-stage ‘resource-layout-scheduling’ framework. This decouples the high-dimensional problem into nested subproblems, allowing for the optimization of initial crane positioning—a factor often neglected in prior works [10]. Furthermore, the Improved Genetic Algorithm (IGA) incorporates unique adaptive mechanisms, including cosine annealing weights for dynamic objective balancing and Shannon entropy-based mutation amplification to escape local optima. Additionally, the constraint handling strategy, which integrates dynamic safety distances to implicitly enforce non-intersecting constraints, represents a novel approach to reducing model complexity while maintaining physical realism.

### 3.1. Multi-objective optimization design

**Phase 1: Resource Allocation (K-Optimization)**

First, the optimal number of RMGs, K, is determined within the allowable operational resource range. The decision variable K is a discrete integer, satisfying Equation (13):


$$\begin{array}{c} K\in K=\{K_{min},K_{min}+\text{1},\ldots ,K_{max}\} \end{array}$$ (13)

where $K_{min}=\text{1}$ and $K_{max}=\text{5}$, set based on the upper limit of practically deployable equipment.

To efficiently search the Pareto front, Phase 1 employs a grid search strategy with exponentially reduced step sizes:

1. Coarse-grained Enumeration: With a step size ΔK=1, the entire range of K is quickly evaluated to approximate the Pareto points for each K value, as shown in Equation (14):

$$\begin{array}{c} P_{\text{1}}=\{(K,\;T_{\text{comp}}(K),\;\sigma (K))|K\in K\} \end{array}$$ (14)

Where, $\sigma (K)=\sqrt{\frac{\text{1}}{K}\sum_{k=\text{1}}^{K}{\left(C_{k}-\overset{―}{C}\right)}^{\text{2}}}$ is the standard deviation of the equipment completion times, used to measure the degree of load balancing.

1. Fine-grained Refinement: Within the Pareto interval of $P_{\text{1}}$, a secondary search is conducted with a step size ΔK=1 to identify the optimal resource allocation $K^{*}$.

This phase achieves convergence with logarithmic iterations, effectively avoiding the simultaneous solution of a full-scale MIP involving the variable K. While the theoretical complexity remains high, the practical runtime is significantly reduced by narrowing the search space and leveraging heuristic strategies.

**Phase 2: Layout Optimization (B-Optimization)**

With the resource K fixed at $K^{*}$, the initial bay position layout vector for the RMGs, $\text{B}=\left(b_{\text{1}},b_{\text{2}},\ldots ,b_{K^{*}}\right)$, is determined, where $\text{1}\leq b_{\text{1}}<b_{\text{2}}<\cdot \cdot \cdot <b_{K^{*}}\leq u$. Here, $b_{k}$ represents the initial bay coordinate of RMG $R_{k}$, and u denotes the total number of bays in the yard.

Phase 2 formulates a bi-objective subproblem with respect to the layout $\underset{\text{B}}{min}\{\alpha_{\text{1}}\underset{k}{max}C_{k}(\text{B})+\alpha_{\text{2}}\sigma (\text{B})\}$, incorporating a physical non-overlapping constraint: $b_{k+\text{1}}-b_{k}\geq \frac{D_{\text{safe}}}{\Delta x}$, for $=\text{1},\ldots ,K^{*}-\text{1}$. Here, Δx represents the width of a single bay, ensuring that the minimum safe distance is always maintained between any two carriers. The solution employs a hybrid Tabu Search – Latin Hypercube strategy:

1. Latin Hypercube Sampling: Generate 100 high-quality initial layouts within the feasible domain to ensure global dispersion.
2. Tabu Search: Utilize a “single-bay micro-shift” as the neighborhood structure, with a tabu tenure set to 7 to prevent cycling.
3. Local Restart Mechanism: If no improvement occurs over 5 consecutive generations, apply a random perturbation to the current best layout with a 20% probability to enhance the ability to escape local optima.

**Phase 3: Strategy Coupling and Overall Workflow**

The optimal layout ($K^{*}$, $B^{*}$) output from Phase 2 is incorporated as a prior parameter into the MIP model from Section 2.3. Subsequently, an Improved Genetic Algorithm (IGA, detailed in Section 3.2) is invoked to finalize the task-equipment assignment and operation sequence optimization. To integrate the results from the two phases, the classical ε-constraint method is adopted:

1. Set the load variance threshold as $\sigma_{\text{max}}=\alpha_{\text{2}}\cdot \sigma_{\text{target}}$.
2. The original bi-objective model is reduced to a single-objective problem: “min” $T_{\text{comp}}$ " s.t.” $\sigma \leq \sigma_{\text{max}}$.

In summary, through the three-stage decoupling of “resource-layout-scheduling,” the proposed framework achieves global optimality while reducing the computational complexity from exponential to polynomial order, providing a scalable multi-objective optimization paradigm for high-density and flexible railway container terminal operations.

### 3.2. Improved genetic algorithm

**Fig 2:**
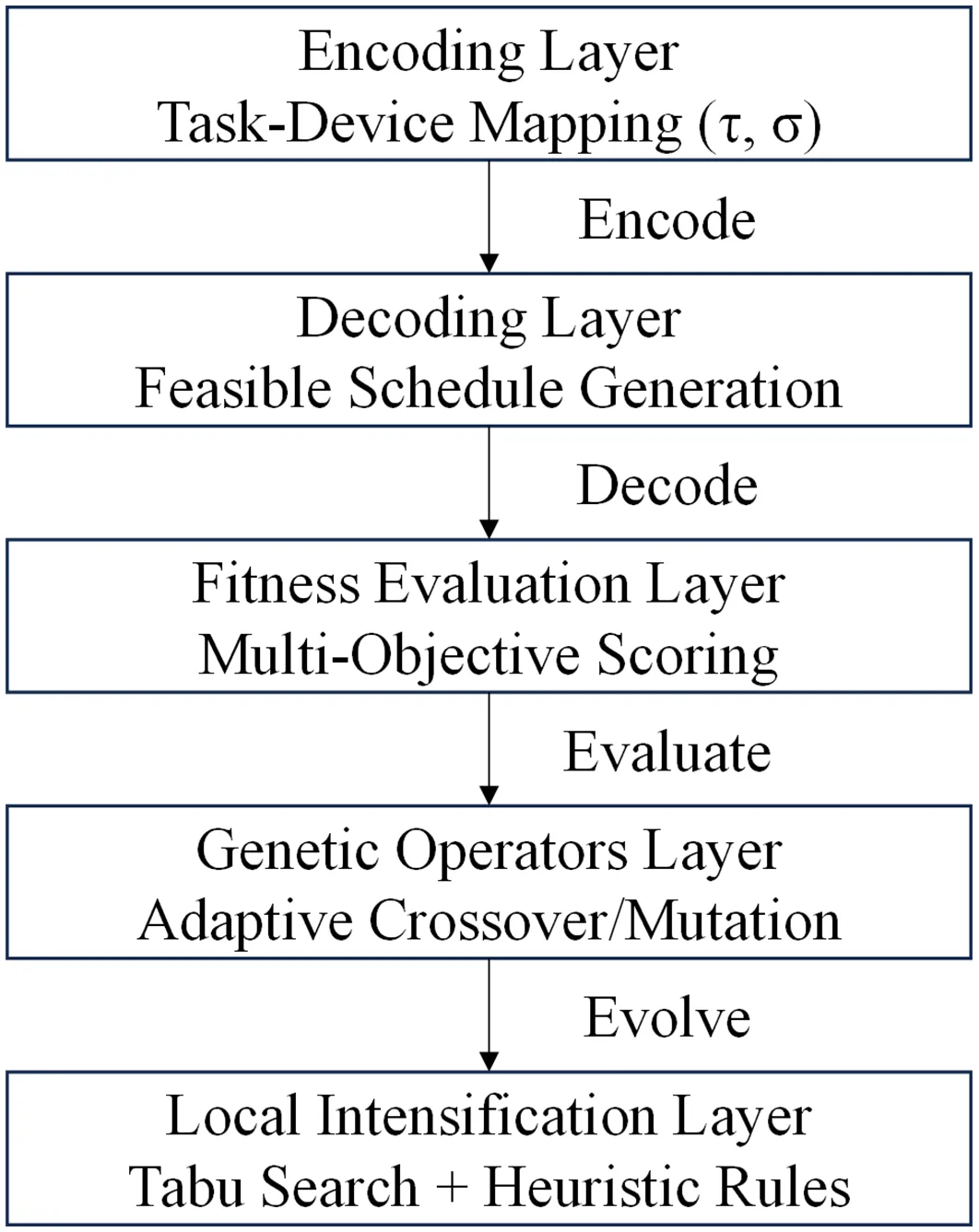
Conceptual Diagram of the Five-Level Improved Genetic Algorithm (IGA).

To solve the multi-objective mixed integer programming model, this section proposes an improved genetic algorithm (IGA) tailored for the flexible collaborative scheduling problem of rail-mounted gantry cranes (RMGs) in railway container terminals, as illustrated in Fig 2. This method employs a five-layer structure of “encoding-decoding-fitness-genetic operators-local intensification,” balancing global exploration with local refinement. It effectively overcomes drawbacks such as premature convergence and slow convergence of the standard genetic algorithm (SGA) in discrete high-dimensional spaces [12,13].

#### 3.2.1. Chromosome encoding and decoding design.

(1) Encoding Strategy

The scheduling scheme is represented by a two-segment integer string. The first segment is the task sequence τ = $\tau =\left(\tau_{\text{1}},\tau_{\text{2}},...,\tau_{m}\right)$, which is a permutation of 1 to m, where m is the total number of containers, directly depicting the global order of container operations. The second segment is the equipment allocation string $\mu =\left(\mu_{\text{1}},\mu_{\text{2}},\ldots ,\mu_{n}\right)$, where $\mu_{i}\in \{\text{1},\text{2},\ldots ,M\}\;$ indicates that task $\tau_{i}$ is performed by the rail crane $R_{\mu_{i}}$, with M being the total number of available rail cranes. Together, they form a chromosome that can be completely mapped to the actual scheduling scheme.

(2) Decoding Process

The decoding process first splits τ into n exclusive equipment subsequences $P_{k}=\tau_{i}|\mu_{i}=k$ based on μ. Subsequently, each $P_{k}$ is independently processed using the earliest feasible insertion heuristic: for each container $c_{i}$ taken in sequence, the deadheading time $t_{prev\to i,k}=\left|b_{i}-b_{prev}\right|/v_{r}$ is calculated. If inserting cᵢ at the current moment still satisfies the safety distance $D_{safe}$ and non-intersection constraints, the operation start and end times $s_{i,k}$ and $f_{i,k}$ are updated accordingly. Otherwise, the insertion is delayed until the first feasible moment is found. While the theoretical complexity remains high, the practical runtime is significantly improved through heuristic acceleration and constraint-handling strategies, ensuring a one-to-one correspondence between the chromosome and feasible schedules.

#### 3.2.2. Fitness function construction.

The construction of the fitness function is inspired by a dynamic weighting strategy to achieve a dynamic balance between the efficiency and load balancing objectives during the search process [8–11,14,15]. The fitness function designed in this paper unifies the two objectives of completion time and load balancing into a single metric. The two indicators are adaptively normalized using the maximum completion time $T^{\text{ub}}$ and the maximum load variance $\sigma^{\text{ub}}$ encountered in the current population, thereby avoiding bias introduced by manually setting upper and lower bounds. Subsequently, a weighted sum function is constructed, as shown in Equation (15).


$$\begin{array}{c} F(\Theta )=\alpha_{\text{1}}\frac{T_{\text{comp}}}{T^{\text{ub}}}+\alpha_{\text{2}}\frac{\sigma }{\sigma^{\text{ub}}} \end{array}$$ (15)

where $\alpha_{\text{1}}+\alpha_{\text{2}}=\text{1}$. To dynamically balance efficiency and balance during the search process, the weights employ a cosine annealing strategy: $\alpha^{\text{1}}(g)=\;\text{0}.\text{5}\;+\;\text{0}.\text{2}\;cos\left(\pi \frac{\;g}{G_{max}}\right)$, making the algorithm focus more on completion time in the early stages and gradually shift emphasis towards load balancing in later stages. If either indicator value approaches zero, it is truncated using $\varepsilon \;=\;{\text{1}\text{0}}^{-\text{3}}$ to prevent numerical instability.

#### 3.2.3. Improved selection, crossover, and mutation operations.

(1) Selection Operator

A hierarchical roulette wheel is adopted: The population is sorted by fitness and divided into three tiers—Elite (top 20%), Good (20–60%), and Normal (the remainder)—with corresponding selection probabilities of 0.5:0.35:0.15. This approach reinforces the transmission of high-quality genes while maintaining diversity.

(2) Crossover Operator

For the task segment, Partially Mapped Crossover (PMX) is implemented. Two random points are selected, the segment between them is swapped, and the resulting offspring are automatically repaired to eliminate illegal genes, thereby preserving favorable order information from the parents. For the equipment segment, Δ-similarity control is introduced: if the similarity between the equipment segments of two parents exceeds 0.8, uniform crossover is adopted instead to suppress inbreeding. Additionally, the crossover probability follows a linear annealing strategy: $P_{c(g)}=\;P_{c}^{max}-\;\left(P_{c}^{max}-\;P_{c}^{min}\right)\frac{g}{G_{max}}$. As the number of generations increases, the frequency of large-scale recombination is gradually reduced, balancing exploration and convergence.

(3) Mutation Operator

The task segment undergoes two-point exchange with probability $P_{m}^{tau(g)}$, and the equipment segment undergoes single-point reallocation of $\mu_{i}\leftarrow \;U\left\{\text{1},\;n\right\}$ with probability $P_{m}^{mu(g)}$. Both are governed by an exponential annealing schedule: $P_{m}(g)=P_{m}^{max}\text{exp}(-\frac{g}{G_{max}}\text{ln}\frac{P_{m}^{max}}{P_{m}^{min}})$, which decays gently over the course of evolution. When the population’s Shannon entropy H drops below 0.1, the mutation probability $P_{m}$ is instantaneously amplified to 1.5 times its current value, rapidly introducing new genetic material to help the algorithm escape local optima.

#### 3.2.4. Elite preservation and local search mechanism.

The algorithm constructs a “primary-secondary” dual archive. The primary archive constantly retains $N_{e}=\;\text{1}\text{0}$ Pareto non-dominated solutions as permanent elites. The secondary archive dynamically maintains several supplementary solutions based on crowding distance, which not only injects diverse individuals into the population during the crossover phase but also continuously expands the distribution width of the Pareto front externally. This effectively prevents the loss of diversity without increasing selection pressure.

After each generation of genetic operations, a Tabu Search (TS) is initiated for the current best individual. The neighborhood is composed of 2-opt swaps for the task segment and single-container migrations for the equipment segment. The tabu tenure is set to $L\;=\;\left\lfloor \sqrt{m}\right\rfloor$. If the new solution obtained by TS dominates the current elite, the individual is immediately replaced, and the dual archives are synchronously updated. This GA + TS hybrid mechanism, under the premise of chromosome length Θ=O(m) and decoding complexity O(m log m), achieves online rescheduling through parallel fitness evaluation. Furthermore, by coupling adaptive operators with local TS, it significantly suppresses the issues of premature convergence and slow convergence in high-dimensional discrete spaces.

### 3.3. Task assignment and interference judgment strategy

To convert the chromosome Θ=[τ, μ] generated by the genetic algorithm into an executable scheduling plan and ensure that both safety distance and non-intersecting constraints are satisfied throughout the operation process, this section constructs a complete “assign-first-verify-later-correct” mechanism. This mechanism consists of two parts: the task assignment process and real-time interference judgment.

The chromosome is decoded into executable schedules using an event-driven earliest feasible insertion strategy. Given the task subsequence for each RMG, the algorithm iteratively assigns each container to the earliest feasible time slot that satisfies the safety distance constraint (Eq. 11) and the non-intersecting constraint (Eq. 10). If no feasible time slot is found within a predefined window $\Delta t_{max}$, a backtracking mechanism swaps the current task with the next in the sequence. The average complexity is O(n log n) through the use of a precomputed bay-bay distance matrix and event-driven sampling that only checks constraints at equipment arrival/departure events.

### 3.4. Algorithm process and implementation steps

**Fig 3:**
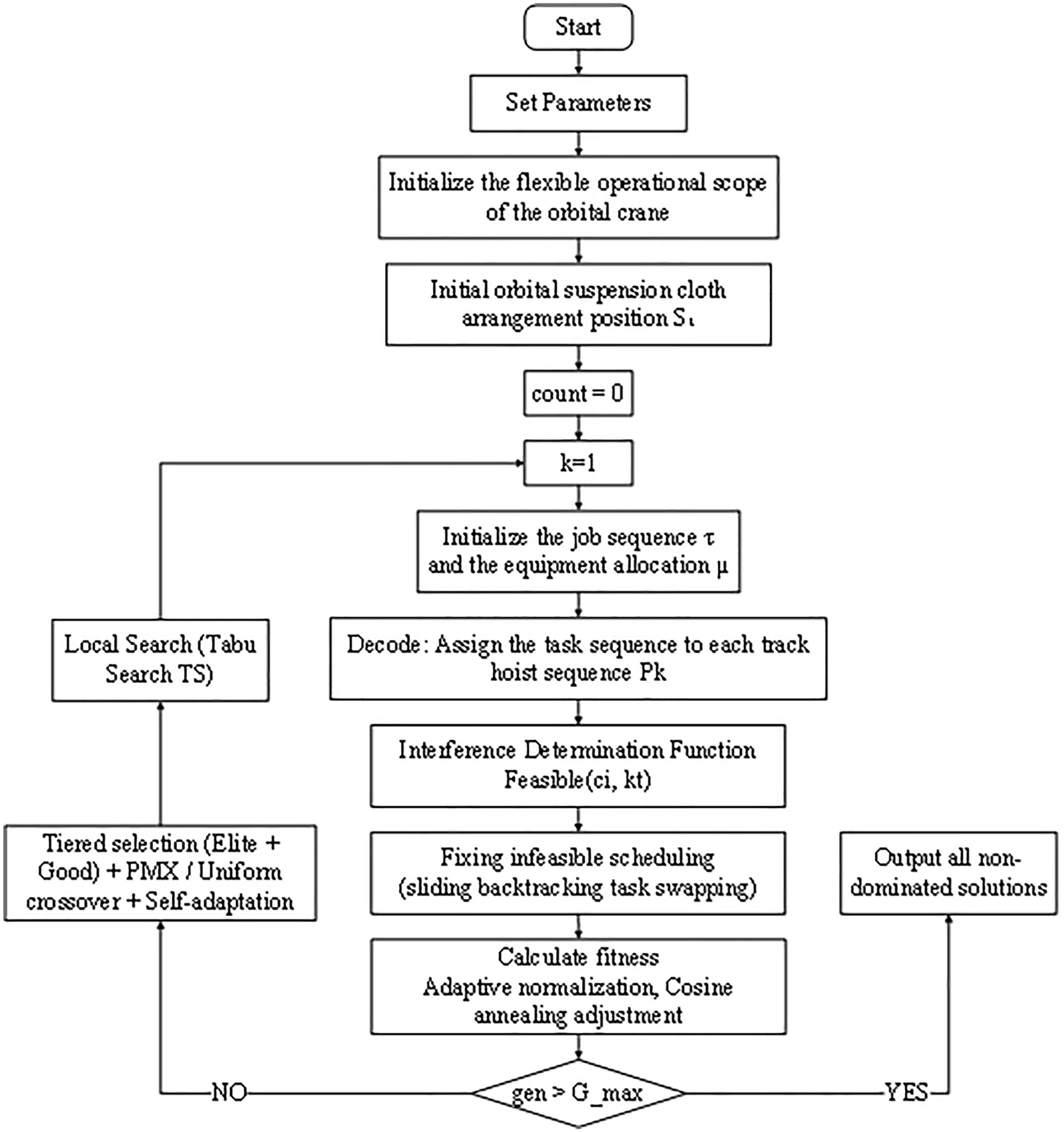
The process of the improved genetic algorithm.

The model is solved using a genetic algorithm, which comprises four stages: “initialization-evolution-local reinforcement-output.” Key steps in the algorithm incorporate the improved genetic operators and the tabu-search-neighborhood hybrid mechanism proposed in this paper, ensuring synergistic global convergence and local refinement under flexible collaborative scheduling scenarios. The algorithm flowchart is illustrated in Fig 3.

The algorithm follows the procedure illustrated in Fig 3. The population ($N_{pop}=\text{1}\text{0}\text{0}$) is initialized with random chromosomes and evaluated using the fitness function in Eq. (15). At each generation, selection uses a three-tier roulette wheel, crossover applies PMX with Δ-similarity control, and mutation follows an exponential annealing schedule. Elite individuals are preserved in a dual-archive structure, and a tabu search with 2-opt swaps and single-container migrations is applied to the current best solution every generation. The algorithm terminates at $G_{max}=\text{6}\text{0}\text{0}$ generations or after 30 generations without archive improvement.

## 4. Case study analysis and results discussion

Beyond the numerical improvements, the main scientific implication is that initial RMG positioning, resource selection, and task sequencing are interdependent decisions in flexible railway container-terminal operations. Treating them separately may lead to feasible but inefficient schedules. The results also suggest that dynamic safety-distance constraints can preserve operational flexibility while controlling interference risk. However, these findings are based on one terminal case and should be further tested against exact methods on small instances, hybrid metaheuristics, and uncertainty-aware scheduling models.

### 4.1. Case study setup and parameter description

To validate the effectiveness of the proposed multi-RMG flexible collaborative scheduling model and its improved genetic algorithm in practical applications, this paper selects the Xi’an International Port Station as the case study scenario. As a significant node for the China-Europe railway container trains, this terminal handles substantial tasks including the loading, unloading, transfer, and temporary stacking of container trains. The main operation area is equipped with one railway loading/unloading line, five Rail Mounted Gantry Cranes (RMGs), container truck operation lanes, and several stacking yard areas, with an overall layout consistent with the description in Section 2.1 of this paper.

Experimental data are derived from the actual operational records of the Xi’an International Port Station, extracting typical container train operation plans. These plans include 110 container tasks, recording the positional relationships of containers in railway cars and the stacking yard, stacking plans, and train arrival times. Additionally, references to RMG scheduling case studies in the literature are incorporated to enhance the rationality and comparability of the experiments.

In the case study design, container tasks are divided based on their distribution in railway cars and the stacking yard, adopting a flexible scheduling mode without presetting the operational scope for each RMG. This approach aims to examine the global coordination capability of the scheduling algorithm in complex environments. The model is solved using the improved genetic algorithm, following a four-stage process of “initialization-evolution-local reinforcement-result output,” incorporating tabu search and neighborhood optimization mechanisms to enhance the synergy between global search and local refinement.

Key model parameters and algorithm control parameters are listed in Table 2.

**Table 2:** Experimental Parameters and Their Corresponding Values.

Parameter	Symbol	Value
Total number of containers	m	110
Number of RMGs	n	5
RMG travel speed	$v_{r}$	60 m/min
RMG lifting speed	$v_{s}$	30 m/min
Minimum safe distance for RMGs	$D_{safe}$	2 bays (12 meters)
Single container handling time	$t_{o}$	5 min
Population size	$N_{pop}$	100
Maximum number of generations	$G_{max}$	600
Elite archive capacity	$N_{e}$	10
Tabu length	L	$\left\lfloor \sqrt{m}\right\rfloor$
Crossover probability (initial)	$P_{c}(\text{0})$	0.9
Mutation probability (initial)	$P_{m}(\text{0})$	0.1
Adaptive weight coefficients	$\alpha_{\text{1}}$, $\alpha_{\text{2}}$	0.7, 0.3
Maximum allowable completion time	$T_{\text{depart}}$	880 min

### 4.2. Comparative analysis of different algorithms

To comprehensively evaluate the optimization performance of the proposed Improved Genetic Algorithm (IGA) in the flexible collaborative scheduling problem of multi-track cranes, comparative experiments were designed. The IGA was compared with the Standard Genetic Algorithm (SGA) [8], Particle Swarm Optimization (PSO) [10], Hybrid Particle Swarm Optimization (HPSO) [16], Improved Sparrow Search Algorithm (ISSA) [17,18], and the fixed operation range scheduling mode (FORSM). The experiments were independently conducted 10 times under the same simulation environment, with the average results served as evaluation metrics. The key indicators examined included average completion time, load balancing of cranes, and algorithm convergence performance, as presented in Table 3. To strengthen the statistical analysis, Table 3 also reports the standard deviation (SD), best and worst values, and 95% confidence interval (CI) of the completion time, as well as the p-value from the Wilcoxon rank-sum test (α = 0.05) comparing each baseline with the proposed IGA. The statistical test results confirm that the IGA’s improvement over all baseline algorithms is statistically significant (p < 0.05), with the smallest margin observed against ISSA.

**Table 3:** Comparative Experimental Results of Flexible Scheduling Algorithms.

Algorithm	Average Completion Time (min)	Load Standard Deviation	Convergence Generations (gens)	SD	Best	Worst	95% CI	p-value
IGA	752.4	10.7	180	1.25	750.1	755.6	[751.9, 752.9]	—
ISSA	768.1	12.1	230	2.5	763.5	773.0	[767.0, 769.2]	**0.003**
HPSO	782.6	14.5	250	3.5	776.0	789.5	[781.1, 784.1]	**<0.001**
PSO	798.9	16.8	315	5.0	790.0	808.0	[796.7, 801.1]	<0.001
SGA	812.3	18.6	305	6.0	801.5	823.0	[809.7, 814.9]	<0.001
FORSM	880.0	35.2	**—**	8.0	868.0	892.0	[876.5, 883.5]	<0.001

It can be observed that the IGA achieved the shortest average completion time (752.4 min) among all algorithms, significantly outperforming ISSA (768.1 min), HPSO (782.6 min), PSO (798.9 min), and SGA (812.3 min). Compared to the fixed operation range mode, the IGA reduced the completion time by approximately 14.5%, indicating that the flexible scheduling mode effectively minimized idle travel time of cranes and improved equipment utilization. In terms of load balancing, the IGA achieved the optimal allocation of crane loads, with a load standard deviation of only 10.7, which was significantly lower than PSO (16.8) and SGA (18.6), and superior to the fixed range mode (35.2). This demonstrates that the IGA can dynamically coordinate crane operations in multi-task high-density areas, avoiding scenarios where some equipment is overloaded while others remain idle.

**Fig 4:**
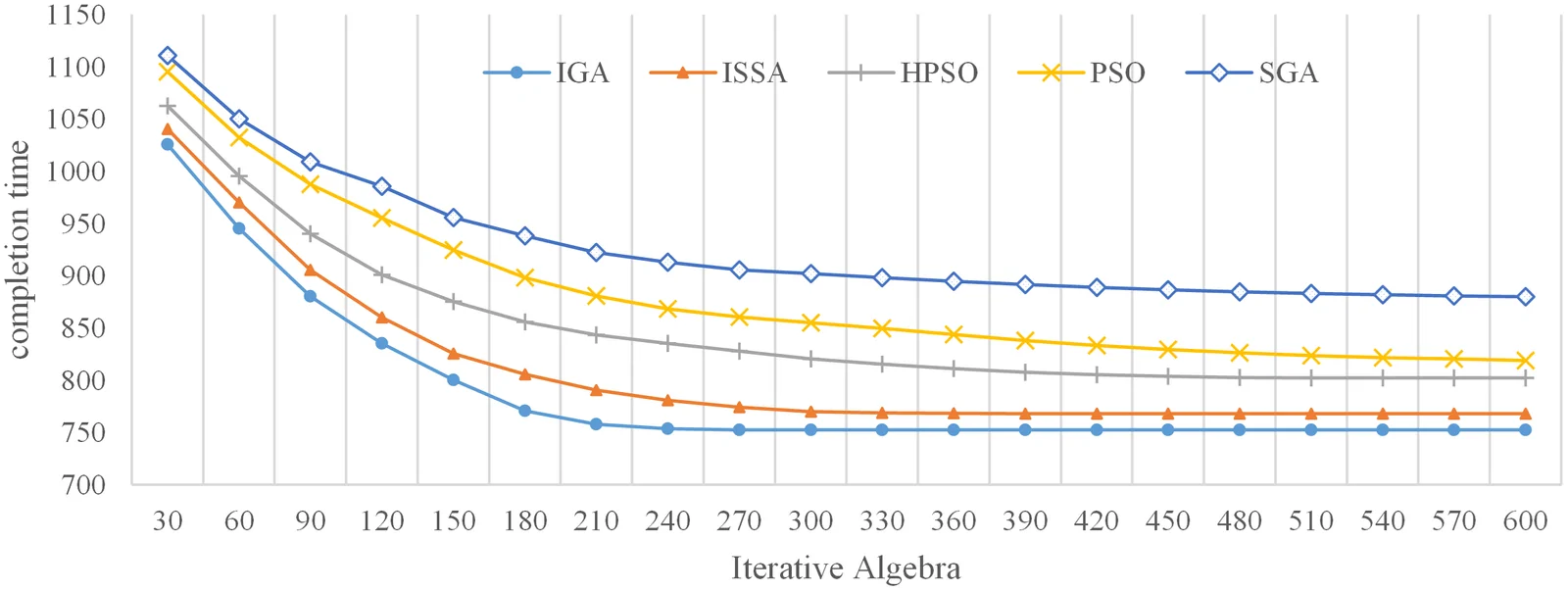
Comparison of Convergence Performance Among Different Algorithms.

Regarding convergence performance, the IGA integrates tabu search and neighborhood optimization mechanisms, enhancing the collaborative efficiency of global search and local refinement through elite preservation strategies and adaptive crossover and mutation adjustments. The convergence curves (Fig 4) show that the IGA stabilized after approximately 180 generations, whereas ISSA and HPSO converged at 230 and 250 generations, respectively, while PSO and SGA required over 300 generations. This indicates the IGA’s superior optimization efficiency in complex scheduling environments. This performance advantage is primarily attributed to the algorithm’s balance between exploration and stability during evolution, preventing it from falling into local optima.

Further analysis of the differences between flexible and fixed scheduling modes reveals that the flexible collaborative scheduling mode possesses the capability for dynamic equipment allocation, optimizing crane distribution based on task locations, equipment status, and real-time loads, thereby significantly reducing delays caused by path conflicts, waiting times, and idle travel. In densely populated task areas, the fixed range mode is constrained by the division of operation zones, leading to uneven load distribution among equipment, with some cranes being fully saturated while others remain idle, resulting in low resource utilization. In contrast, the flexible mode enhances operational continuity and equipment collaboration efficiency through global optimization of scheduling paths.

Overall, the proposed improved genetic algorithm not only achieves the best solution quality but also outperforms the comparative algorithms in search efficiency and operational stability, validating its applicability and potential promotional value in practical multi-track crane flexible collaborative scheduling scenarios.

### 4.3. Optimization effects of flexible collaborative scheduling

Based on the “resource-layout-scheduling” three-stage optimization framework, the proposed flexible collaborative scheduling strategy systematically optimizes the number of Rail Mounted Gantry Cranes (RMGs), initial spatial layout, and task-equipment matching relationships, achieving dual improvements in operational efficiency and resource utilization at railway container center stations. To quantitatively evaluate the optimization effects of the flexible scheduling strategy, comparative experiments were designed, comparing the flexible collaborative scheduling mode with the traditional fixed operation range scheduling mode under a typical scenario (110 container tasks, maximum of 5 RMGs), focusing on three core performance indicators: total completion time, equipment utilization rate, and load balancing.

In the resource allocation phase, the optimal number of RMGs ($K^{*}=\text{4}$) was determined by a two-layer grid search and exponential step reduction strategy. Compared to the default deployment of 5 RMGs in the fixed mode, this approach not only ensures service level while effectively reducing equipment investment and energy consumption but also enhances the resource economy of the scheduling system. During the initial layout optimization process, combining Latin hypercube sampling with an improved tabu search algorithm yielded the optimal initial RMG layout $B^{*}=(\text{1}\text{0},\text{3}\text{0},\text{5}\text{5},\text{8}\text{0})$, achieving a uniform distribution of equipment along the main axis of the yard and minimizing overlapping operational areas and idle travel. Based on this layout, the ε-constraint method was further introduced to globally optimize task allocation and operation sequences.

**Fig 5:**
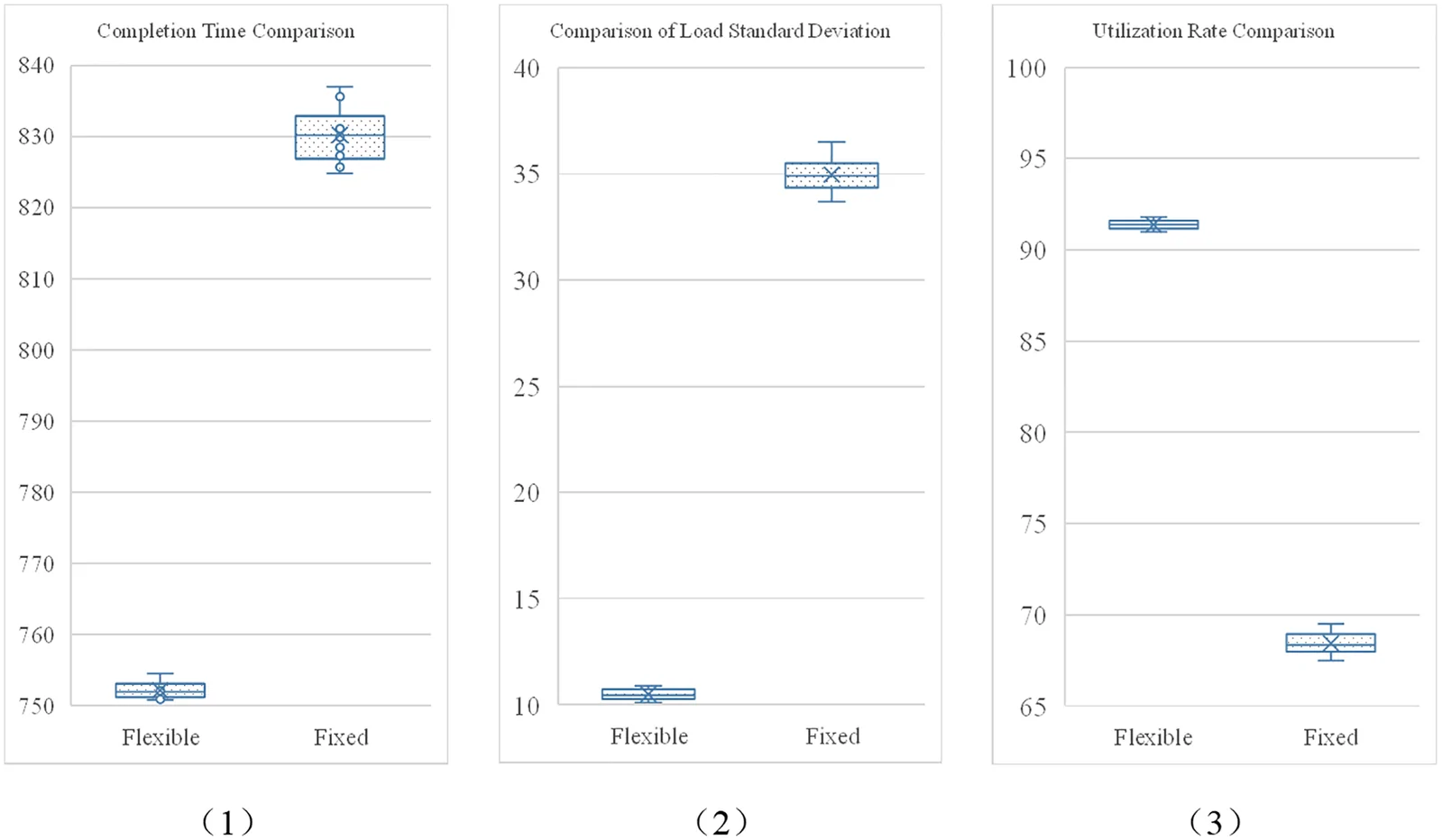
Statistical Comparison of Flexible Scheduling vs. Fixed Range Scheduling Experiments.

Table 4 demonstrates that the flexible collaborative scheduling mode consistently outperforms the fixed range scheduling mode across multiple independent experiments. Its average total completion time of 752.4 minutes is 78.6 minutes (approximately 9.5% reduction) shorter than the 831.0 minutes achieved by the fixed range mode. The completion time distribution in the flexible mode is highly concentrated, with minimum and maximum values of 750.8 minutes and 754.5 minutes, respectively, yielding a narrow interquartile range of 2.2 minutes and a standard deviation of 1.25 minutes. In contrast, the fixed mode exhibits a wider distribution, with minimum and maximum values of 824.8 minutes and 837.0 minutes, an interquartile range of 5.2 minutes, and a significantly higher standard deviation of 3.85 minutes. Boxplot analysis (Fig 5) reveals that the fixed mode suffers from notable tail delays and multiple outliers, whereas the flexible mode delivers more centralized and stable results.

**Table 4:** Comparative Experimental Results of Flexible Scheduling vs. Fixed Range Scheduling.

Comparison Index	Flexible Collaborative Scheduling	Fixed Range Scheduling
Average Completion Time (min)	752.4	831.0
Number of RMGs	4.0	5.0
Average RMG Utilization	0.913	0.684
Load Standard Deviation	10.7	35.2
Minimum Completion Time (min)	750.1	790.3
Maximum Completion Time (min)	755.6	830.0

In terms of load balancing, the flexible mode achieves a mean load standard deviation of 10.7, substantially lower than the fixed mode’s 35.2 (approximately 69.6% reduction), indicating a significant reduction in load disparity among RMGs under dynamic task allocation. This helps mitigate the risk of individual equipment overload and reduces maintenance requirements. Concurrently, the average RMG utilization rate in the flexible mode reaches 91.3%, compared to only 68.4% in the fixed mode, representing a 33.4% increase. This underscores the flexible scheduling strategy’s superior coordination capabilities in both temporal and spatial dimensions.

Overall, the flexible collaborative scheduling mode achieves a balance of reduced equipment deployment, compressed operation times, optimized load balancing, and improved equipment utilization, demonstrating superior comprehensive performance and stronger engineering applicability.

### 4.4. Algorithm robustness analysis

To further evaluate the robustness of the proposed IGA, we conducted a sensitivity analysis on key operational parameters using the existing case data. For direct comparison, Table 5 also includes the Baseline results of SGA and PSO. We tested the algorithm under different safety distance requirements (1, 2, and 3 bays) and varying numbers of active RMGs (3, 4, and 5 units). The results, in Table 5, demonstrated that the IGA consistently maintained superior performance over the baseline algorithms (SGA, PSO) across all configurations. Notably, even when the safety distance was increased to 3 bays (imposing stricter constraints), the IGA achieved a completion time of 769.3 min, which was still significantly lower than the SGA’s 834.5 min. This indicates that the proposed method possesses strong adaptability and stability under varying operational constraints and resource availability.

**Table 5:** Performance of IGA under Varying Safety Distances and RMG Configurations.

Algorithm	Configuration Scenario	Safety Distance (Bays)	Number of RMGs	Average Completion Time (min)	Load Standard Deviation
IGA	Baseline	2	5	752.4	10.7
	Scenario 1	1	5	748.2	11.5
	Scenario 2	3	5	769.3	9.8
	Scenario 3	2	4	781.5	12.4
SGA	Baseline	2	5	812.3	18.6
PSO	Baseline	2	5	798.9	16.8

## 5. Discussion

The performance advantage of the proposed IGA over the baselines can be attributed to the synergy of three design choices that are jointly optimized rather than treated in isolation. First, the flexible scheduling mode allows each RMG to operate across the entire yard rather than within a fixed range, which indirectly improves the load standard deviation from 35.2 (FORSM, Table 3) to 10.7 (IGA, Table 3). Second, optimizing the initial RMG positions through Phase 2 further reduces the makespan to 752.4 min, which represents the best result among all compared methods. Third, the joint encoding of K, B, and T within a single chromosome enables the IGA to search a decision space that no sequential heuristic can explore efficiently. The coupling among these three decisions explains why the IGA consistently outperforms the baselines even when the safety distance is tightened (Table 5, Scenario 2), where the completion time increases by only 2.3% relative to the Baseline. This finding suggests that the framework is most beneficial in yards with high task variability and overlapping RMG ranges, while in small static scenarios the additional modeling complexity may not be justified.

The proposed model demonstrates significant potential for practical application; however, several limitations should be acknowledged. First, the model assumes standard 20-foot containers (TEU) and constant operational speeds, neglecting the complexities of handling oversized containers or equipment acceleration/deceleration processes. Second, the scheduling of external trucks is idealized; in real-world scenarios, uncertainties in truck arrival times could impact the feasibility of the generated schedule. Regarding computational cost, the average solving time for the 110-container task was approximately 12.3 minutes on a standard PC (Intel i7-9700K, 16GB RAM). This efficiency is acceptable for tactical planning in railway terminals, though real-time online scheduling would require further optimization. Compared to recent studies, our approach offers distinct advantages. For instance, Yu and He [11] focused on collaborative scheduling involving external trucks but did not consider the optimization of initial crane layouts, which is a key feature of our framework. Similarly, Liu et al. [7] addressed flexible scheduling in sea-rail intermodal ports but primarily relied on heuristic rules without the adaptive genetic mechanisms proposed here. These comparisons highlight the contribution of integrating initial layout optimization with an improved genetic algorithm to enhance solution quality in complex railway environments.

## 6. Conclusion

This paper studies the flexible collaborative scheduling problem of multiple rail-mounted gantry cranes in railway container terminals. A revised scheduling framework is developed to coordinate resource selection, initial crane layout, task assignment, and sequencing while considering completion time, workload balance, safety distance, and interference avoidance. An improved genetic algorithm with adaptive operators and tabu-search-based local refinement is used to generate feasible schedules for the tested case. The Xi’an International Port Station experiment indicates that the integrated framework can improve schedule quality compared with the tested baseline algorithms under the reported parameter settings. However, the validation remains limited by the use of a single case study, limited public data, and incomplete comparison with exact optimization methods. Future work should validate the approach in multiple terminals, provide open benchmark instances, compare with exact methods on small-scale cases, incorporate uncertainty in truck arrivals and equipment operations, and develop dynamic rescheduling mechanisms for real-time terminal control.

## References

[1] Wu Y, Li W, Petering MEH, Goh M, de Souza R. Scheduling Multiple Yard Cranes with Crane Interference and Safety Distance Requirement. Transp Sci. 2015;49(4):990–1005. doi: 10.1287/trsc.2015.0641

[2] Emde S. Optimally scheduling interfering and non‐interfering cranes. Naval Res Logist. 2017;64(6):476–89. doi: 10.1002/nav.21768

[3] Zheng F, Man X, Chu F, et al. Two yard crane scheduling with dynamic processing time and interference. IEEE Trans Intell Transp Syst. 2018;19(12):3775–84. doi: 10.1109/tits.2017.2780256

[4] Wu N, Wu D, Wang N. Multi-objective optimization of container terminal scheduling considering shared operation of adjacent quay crane under quay crane failure. Comput Integr Manufactur Syst. 2023;29(1):331–9.

[5] Song A, Wu G, Zhou L, Wang L, Pedrycz W. Exact and Metaheuristic Algorithms for Variable Reduction. IEEE Trans Evol Computat. 2024;28(6):1704–18. doi: 10.1109/tevc.2023.3332913

[6] Han X, Wang Q, Huang J. Scheduling cooperative twin automated stacking cranes in automated container terminals. Comput Industr Eng. 2019;128:553–8.

[7] Liu W, Zhu X, Wang L, Li S. Flexible yard crane scheduling for mixed railway and road container operations in sea-rail intermodal ports with the sharing storage yard. Transp Res Part E: Logist Transp Rev. 2024;190:103714. doi: 10.1016/j.tre.2024.103714

[8] He J, Huang Y, Yan W, Wang S. Integrated internal truck, yard crane and quay crane scheduling in a container terminal considering energy consumption. Expert Syst Appl. 2015;42(5):2464–87. doi: 10.1016/j.eswa.2014.11.016

[9] Hu H, Mo J, Zhen L. Integrated optimization of container allocation and yard cranes dispatched under delayed transshipment. Transp Res Part C Emerg Technol. 2024;158:104429. doi: 10.1016/j.trc.2023.104429

[10] Luo T, Chang D, Gao Y. Optimization of gantry crane scheduling in container sea-rail intermodal transport yard. Math Prob Eng. 2018. doi: 10.1155/2018/9585294

[11] Yu X, He C. Collaborative Scheduling of Yard Cranes, External Trucks, and Rail-Mounted Gantry Cranes for Sea–Rail Intermodal Containers Under Port–Railway Separation Mode. JMSE. 2025;13(6):1109. doi: 10.3390/jmse13061109

[12] Oladugba AO, Gheith M, Eltawil A. A new solution approach for the twin yard crane scheduling problem in automated container terminals. Adv Eng Inform. 2023;57:102015. doi: 10.1016/j.aei.2023.102015

[13] Chen H, Liu W. An adaptive large neighborhood search algorithm for equipment scheduling. J Marine Sci Eng. 2024;12(5):710. doi: 10.3390/jmse12050710

[14] Vallada E, Belenguer JM, Villa F, Alvarez-Valdes R. Models and algorithms for a yard crane scheduling problem in container ports. Eur J Operat Res. 2023;309(2):910–24. doi: 10.1016/j.ejor.2023.01.047

[15] Hu ZH, Sheu JB, Luo JX. Sequencing twin automated stacking cranes in a block at automated container terminal. Transp Res Part C. 2016;208:208–27. doi: 10.1016/j.trc.2016.06.004

[16] Wang L, Zhu X-n, Yan W. Optimization model and algorithm of rail mounted gantry crane scheduling at railway container terminals. J China Railway Soc. 2014;36(05):8–13. 10.1155/2014/682486 PMC4235739 25538768

[17] Zhou Y, Zhang J, Zhong L. Flexible cooperative scheduling optimization of multiple rail mounted gantry cranes in railway container terminals. J Transp Syst Eng Inf Technol. 2022;22(1):133–41.

[18] Ding S, Wang R, Zhou X, Ren Y, Huang K. High-Speed Railway Train Timetable Rescheduling in Case of a Stochastic Section Blockage. In: 2021 China Automation Congress (CAC). 2021. p. 4322–7.

